# Nanotechnology-based drug formulation and delivery systems for skin medical aesthetic dermatology: mechanisms, applications, and safety

**DOI:** 10.3389/fphar.2026.1804123

**Published:** 2026-04-15

**Authors:** Luqi Wang, Debao Yu

**Affiliations:** Department of Medical Aesthetics, Jinan Institute of Dermatology Prevention and Treatment Jinan, Shangdong, China

**Keywords:** aesthetic dermatology, dermaldrug delivery, nanocarriers, nanotechnology, skin penetration, translational pharmacology

## Abstract

Medical aesthetic dermatology is expanding rapidly in response to increasing demand for minimally invasive interventions and advanced topical therapies. The clinical performance of conventional dermatological formulations is frequently limited by inadequate skin penetration, poor stability of active ingredients, rapid degradation, and dose-related adverse effects. Nanotechnology-based drug formulation and delivery systems offer a translational solution to these challenges by enabling targeted, controlled, and sustained delivery of bioactive compounds to specific skin layers and appendages. Nanoscale carriers—including lipid-based systems, polymeric nanoparticles, nanoemulsions, and inorganic or hybrid nanomaterials—enhance dermal and follicular deposition, improve physicochemical stability, and modulate release kinetics of therapeutic and cosmeceutical agents. These mechanisms have facilitated improved outcomes across key aesthetic indications, including skin rejuvenation, pigmentary disorders, acne, hair restoration, and injectable aesthetic interventions. Emerging clinical and preclinical evidence suggests improved local efficacy and potentially reduced irritation and systemic exposure compared with conventional formulations, although further well-controlled clinical studies are required to confirm these benefits. Safety, toxicological behavior, and regulatory considerations remain critical determinants of successful clinical translation, particularly with respect to long-term exposure and nanoparticle–skin interactions. Integration of pharmacological mechanisms with clinical performance highlights the growing role of nanotechnology in redefining therapeutic strategies within medical aesthetic dermatology and supports its continued development within evidence-based translational pharmacology frameworks.

## 1 Introduction

Global demand for aesthetic dermatology is increasing rapidly. It is driven by aging populations and patient interest in non-invasive skin rejuvenation (Liu et al., 2024). The cosmetic dermatology and cosmeceutical market has grown into a multibillion-dollar industry globally. Recent evidence shows that the global nanomaterials market in cosmetics was valued at USD 8.5 billion in 2019 and is projected to grow at ∼13% CAGR through 2027. This growth reflects strong investment and consumer interest. Alongside this growth, the market for advanced cosmetic formulations—such as nanotechnology-enabled sunscreens, anti-aging creams, and antioxidant delivery systems—has expanded rapidly (Latini et al., 2025). By 2020, the cosmetic segment alone contained thousands of nanotechnology-based products (like sunscreens, moisturizers) with improved performance (Cardoza et al., 2022). However, despite this rapid commercial expansion, significant challenges persist in conventional aesthetic therapies that limit their clinical effectiveness. Many topical actives (retinoids, antioxidants, vitamins) show poor skin retention and penetration. Furthermore, they require frequent application and are chemically sensitive or photolabile, thus reducing efficacy (Zhong et al., 2024). Irritation and side effects from solvents or high doses are also common. Conventional creams often deliver only shallow epidermal effects due to the protective stratum corneum. This necessitates invasive procedures (peels, injections) for deeper action (Prajapati et al., 2025).

Nanotechnology has emerged as a powerful strategy to address these limitations. By encapsulation of active ingredients in nanoscale carriers (1–1,000 nm), different studies have demonstrated improved skin deposition, controlled release, and stability of cosmetic actives (Oliveira et al., 2022). Early successes like Dior’s liposome-based anti-aging lotions in the 1980s signified the modern trend. Now, lipid and polymeric nanosystems can act as penetration enhancers, UV screens (TiO_2_, ZnO), and targeted delivery vehicles in aesthetic medicine (Nagpal, 2023). Recent reviews highlight the ability of nanocarriers in bolstering cosmeceutical performance and enables replacement of traditional formulations with nanocosmeceuticals (Gupta et al., 2022).

Despite the growing body of literature on nanotechnology in dermatology and cosmetic science, several recent reviews have primarily focused on material development or formulation strategies rather than integrating mechanistic, clinical, and regulatory perspectives. For example, Schleich and Préat (2012) provided an early overview of nanostructures designed to overcome the skin barrier, with emphasis on transdermal drug delivery mechanisms. More recent analyses, such as those by Zhao et al. (2025) and Yan et al. (2026), have highlighted advances in nanocosmeceuticals and topical nanocarrier systems for skincare applications, particularly focusing on material innovation and formulation design. However, these studies generally emphasize technological development and cosmetic applications without providing a comprehensive translational framework that integrates biological mechanisms of skin penetration, clinical evidence, safety considerations, and regulatory challenges within the field of medical aesthetic dermatology.

The present review addresses this gap by providing an integrated analysis that connects nanocarrier design with mechanistic understanding of dermal delivery, emerging clinical evidence, safety and toxicological considerations, and the evolving regulatory landscape. By linking these interdisciplinary perspectives, this work seeks to provide a more comprehensive translational overview of nanotechnology-based strategies in medical aesthetic dermatology and to highlight key research priorities that may guide future clinical development and regulatory standardization.

The aim of this review is to comprehensively examine the transformation of medical aesthetic dermatology by nanotechnology-based drug delivery systems. Specifically, this review delineates the fundamental mechanisms by which nanoscale carriers improve skin penetration, stability, and controlled release of active compounds. It further evaluates the clinical relevance of these advances across a spectrum of aesthetic indications such as skin rejuvenation, pigmentation disorders, acne management, hair restoration, and injectable therapies. By distinguishing between cosmetic and medical aesthetic applications, the review critically assesses translational potential, regulatory considerations, and safety profiles of various nanocarrier platforms. Hence, this review provides high-level synthesis for clinicians, researchers, and regulatory stakeholders engaged in field of aesthetic nanomedicine.

## 2 Skin structure and barriers to dermal drug delivery

### 2.1 Anatomy and physiology of human skin

The skin is a complex multi-layered organ comprised of epidermis (with its outermost stratum corneum), the dermis, and subcutaneous fat (hypodermis). The epidermis is 0.1–0.2 mm thick and contains keratinocytes in the basal and spinous layers. These culminates in stratum corneum (SC). SC is 10–20 µm thick brick-and-mortar structure of 20–30 layers of anucleate corneocytes. These corneocytes are embedded in lipid matrix (ceramides, cholesterol, fatty acids) (Vestita et al., 2022). The SC is the primary barrier against external agents. Underneath, dermis (1–2 mm thick) provides strength (collagen, elastin) and houses blood vessels, nerves, and adnexal structures (hair follicles, sweat and sebaceous glands). The hypodermis consists of adipose tissue and connective tissue. It offers cushioning and insulation (Zwirner and Hammer, 2024).

**FIGURE 1:**
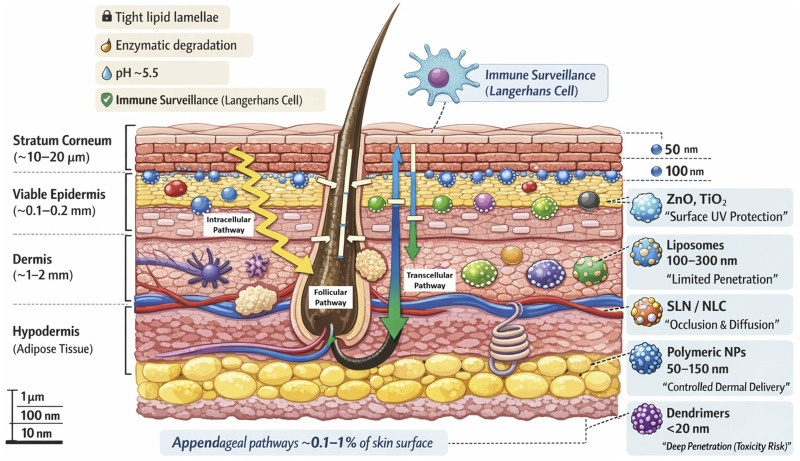
Schematic illustration of human skin layers showing the stratum corneum brick-and-mortar structure, viable epidermis, dermis, and hypodermis, The diagram also illustrates the principal nanoparticle penetration pathways in skin, including the intercellular pathway (between corneocytes), the transcellular pathway (through corneocytes), and the follicular or appendageal pathway via hair follicles and glands.

Beyond these layered structures, specialized skin appendages provide alternative pathways for penetration of nanoparticle and therapeutic targeting. Skin appendages like hair follicles and glands span epidermis and dermis provide alternative penetration pathways. Hair follicles penetrate deep into dermis, are surrounded by sebaceous glands, and contain pluripotent stem cells. These make them target for some nanoparticle delivery. Sweat glands also traverse epidermis and deliver to the surface. Together, appendages comprise approximately 0.1%–1% of skin surface and offer channels for nanoparticle uptake, especially for particulate carriers small enough (typically <100 nm) (Salazar et al., 2022; Peng et al., 2025). The complex architecture of the skin, particularly highly organized stratum corneum and associated appendageal structures is illustrated schematically in Figure 1.

Physiologically, skin acts as a dynamic barrier. It undergoes desquamation (shedding of corneocytes) while metabolically active enzymes in viable epidermis can degrade xenobiotics. It maintains slightly acidic pH (∼5.5) and immunologically surveils intruders (Langerhans cells in the epidermis). These features support homeostasis but complicate topical drug delivery (McKnight et al., 2022).

### 2.2 Challenges in transdermal and intradermal delivery

The formidable skin barrier severely limits the passive penetration of drug. Only small (MW <500 Da) and moderately lipophilic molecules can diffuse through intact SC. Larger, charged, or hydrophilic actives cannot cross the lipid-rich matrix. Moreover, the tight packing of intercellular lipids (ceramides forming lamellae) hinders diffusion. As a result, conventional creams or ointments often confine actives to the epidermal surface or superficial layers (Albakr et al., 2024).

Consequently, researchers have investigated alternative penetration pathways and delivery enhancement strategies. There are multiple penetration routes that exist. The principal pathways are intercellular (through lipid channels between corneocytes) and transcellular (through the cells themselves). These are torturous paths and highly selective based on affinity of lipid (Lee, 2025). Alternatively, appendageal route via hair follicles and sweat ducts bypasses SC to some extent, but is limited by small surface area (Pinnamraju et al., 2025). Nanocarriers can exploit hair follicles as reservoirs, and this follicular targeting represents a key advantages of nanoscale delivery systems compared with traditional topical formulations. Indeed, the hair follicle route is often used for colloidal systems. Finally, transappendageal delivery (through glands) is minor (Kumar et al., 2026).

Different carriers face different penetration fates. Metal oxide sunscreens (TiO_2_, ZnO) aim to remain on SC surface and scatter UV light and avoid deeper absorption. In contrast, carriers for therapeutic delivery (corticosteroids, antioxidants) must traverse to the epidermis/dermis. Excess penetration can lead to systemic absorption and side effects (Liu et al., 2024; Araki and Baby, 2025). For example, hydrocortisone NPs intended for dermal therapy could enter systemic circulation. Thus, tunable penetration is critical (Alsaidan et al., 2023).

Intradermal injection or microneedles bypass the SC barrier altogether, but these are invasive and not ideal for routine aesthetic use. Therefore, most cosmetic/drug delivery relies on the enhancement of transdermal flux. Conventional strategies (chemical penetration enhancers, iontophoresis, abrasion) have limitations (irritation, safety issues, inconsistent results). By comparison, nanotechnology-based carriers offer a hopeful means to overcome barriers without disruption of skin integrity (Jaiswal and Jawade, 2024; Liu et al., 2024).

## 3 Nanotechnology-based delivery platforms in aesthetic dermatology

**FIGURE 2:**
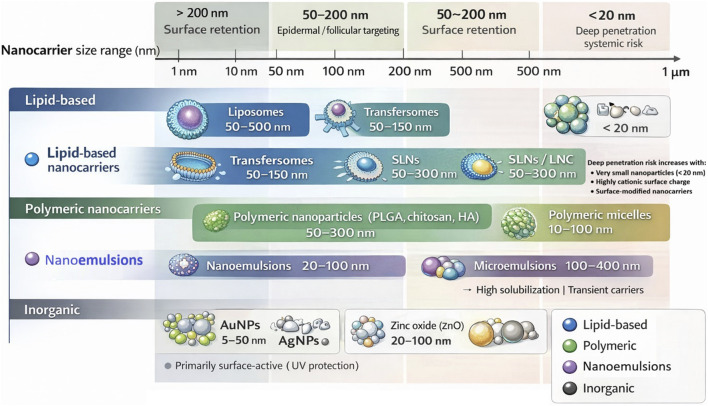
Comparative classification of major nanocarrier systems used in aesthetic dermatology according to their typical size ranges. The schematic also illustrates conceptual functional zones associated with skin surface retention, epidermal or follicular targeting, and potential deep penetration. Increased penetration risk is generally associated with extremely small nanoparticles (<20 nm), highly cationic surface charge, or surface-modified nanocarriers capable of enhanced interaction with skin structures.

A wide variety of nanoscale delivery systems have been developed for skin applications, including lipid nanoparticles, polymeric carriers, nanoemulsions, and hybrid nanomaterials designed to enhance dermal and transdermal drug delivery (Erdoğar et al., 2025; Yan et al., 2026). Broadly, these include lipid-based carriers, polymeric nanoparticles, nano/microemulsions, and inorganic/hybrid nanomaterials (Figure 2).

### 3.1 Lipid-based nanocarriers

#### 3.1.1 Liposomes and niosomes

Liposomes were the first nanoparticulate delivery vehicles and remain extensively studied. They are self-assembled phospholipid bilayer vesicles that entrap aqueous-phase actives inside and lipophilic actives within the bilayer. Liposomes (20 to more than 500 nm in size) enhance skin delivery via multiple mechanisms. The intact vesicles can fuse with SC lipids (horizontal or vertical transport). They serve as penetration enhancers by fluidizing lipids and they form a depot on skin that sustains release (Huang et al., 2024). Modern formulations include ultradeformable transfersomes and ethosomes. Transfersomes are liposomes with edge-activators that squeeze through pores. They have been used for transdermal drug delivery (insulin, NSAIDs) and hold promise for cosmeceuticals (Matharoo et al., 2024). Likewise, ethosomes are liposomes with high ethanol content, which makes them highly fluid. They show enhanced dermal delivery of retinoids and peptides (Musielak and Krajka-Kuźniak, 2024).

In contrast to phospholipid-based vesicles, surfactant-based vesicular systems such as niosomes offer cost-effective alternatives. Niosomes are similar to liposomes but made of non-ionic surfactants and cholesterol. They encapsulate hydrophilic drugs in a central aqueous compartment. Niosomes offer enhanced stability and are cost-effective (no phospholipids). In cosmetics, niosomes have delivered antioxidants and herbal extracts. However, they may be less biocompatible than true liposomes, and surfactant irritancy can be a concern (Mawazi et al., 2022; Kumari et al., 2024).

#### 3.1.2 Solid lipid nanoparticles and nanostructured lipid carriers

Solid lipid nanoparticles (SLNs) are submicron-sized colloidal carriers composed of a solid lipid matrix stabilized by surfactants (Geszke-Moritz and Moritz, 2016). They combine the biocompatibility of lipids with the stability of solids. Typical lipids include triglycerides (glyceryl behenate), fatty acids, or waxes. SLNs (50–300 nm) encapsulate lipophilic actives in a solid core. Advantages include controlled release (the drug slowly diffuses from the solid matrix), good occlusion (improves skin hydration), and UV-blocking (solid lipids scatter light). SLNs protect sensitive ingredients, such as vitamins, from oxidation (Raszewska-Famielec and Flieger, 2022).

Nanostructured Lipid Carriers (NLCs) are an improved SLN. They incorporate a mixture of solid and liquid lipids (oils) to create imperfections in the matrix. This allows higher payloads and prevents expulsion. NLCs maintain the occlusive and protective effects of SLNs, with better stability over time. Both SLNs and NLCs can enhance dermal penetration through occlusive effects that increase skin hydration and alter stratum corneum lipid packing. They create a thin lipid film that increases skin hydration and alters SC lipid packing (Khan et al., 2023; Pareek et al., 2025).

#### 3.1.3 Lipid Nanocapsules, Cubosomes and Glycerosomes

Lipid Nanocapsules (LNCs) are typically a liquid oil core (medium-chain triglyceride) surrounded by a lecithin/surfactant shell. They are smaller (20–100 nm) and very stable. LNCs have been used for topical delivery of lipophilic actives (e.g., sunscreens, antifungals) with the same safety advantages of lipid systems (Rai et al., 2026).

Cubosomes and Glycerosomes are less common cubic-phase lipid nanoparticles. They are lipid bicontinuous cubic phases stabilized by polymers. Cubosomes have a honeycomb structure (20–300 nm) with high internal volume for actives. They show sustained release and good loading of hydrophobic compounds. However, commercial use in cosmetics is still emerging (Dey and Dubey, 2023).

### 3.2 Polymeric nanoparticles

Polymeric nanoparticles encompass a broad class of carriers made from synthetic or natural polymers. They include solid particles (nanospheres/nanocapsules), dendrimers, micelles, and polymer vesicles. Opposite to the lipid carriers, polymeric NPs often use polymers like poly (lactic-co-glycolic acid) (PLGA), polycaprolactone, polystyrene (non-degradable), or natural polymers (chitosan, alginate, gelatin, hyaluronic acid, etc.). Compared to lipids, polymeric NPs generally offer superior structural stability and controllable release kinetics. They also allow higher degrees of functionalization (ligands, antibodies) to target specific skin receptors. On the downside, polymeric NPs may require organic solvents in fabrication, and batch-to-batch reproducibility can be a challenge (Beach et al., 2024; Eltaib, 2025).

#### 3.2.1 Synthetic polymeric NPs

Synthetic polymeric NPs such as PLA, PLGA, PCL are widely used because of tunable biodegradability. PLGA degrades to lactic/glycolic acid and is cleared via metabolism. These NPs can be engineered for sustained release. Drugs are often trapped in polymer matrix or core and released by diffusion/erosion. Polymeric NPs protect sensitive molecules (peptides, growth factors) from enzymatic degradation in skin. Surface modification (PEGylation) can further enhance stability and control skin interaction (Mahar et al., 2023). For example, poly (ethylene glycol)-b-polyester Tyrospheres deliver hydrophobic drugs into skin with low systemic exposure (Michniak-Kohn and Kohn, 2023).

#### 3.2.2 Natural polymeric NPs

Besides synthetic polymers, naturally derived polymers have attracted interest due to their inherent biocompatibility. Chitosan nanoparticles are cationic in nature with an approximate size of 100–200 nm. They improve residence on the skin and have intrinsic anti-microbial/anti-oxidant properties. Chitosan NPs have been used for wound healing and in the reduction of *Cutibacterium acnes*. Alginate and gelatin NPs can encapsulate vitamins and antioxidants for skin repair. These natural systems often form hydrogels spontaneously and make them ideal carriers. Hyaluronic acid (HA) nanogels have also been used for deep moisturization (Babić Radić et al., 2024; Reis et al., 2025).

### 3.3 Nanoemulsions and microemulsions

Nanoemulsions (NE) and microemulsions (ME) are liquid dispersions. They are considered very useful in cosmetics due to their high solubility and easy application on skin. Both are oil-in-water systems (O/W) where lipid droplets are in the nanometer range. Despite their similar composition, nanoemulsions and microemulsions differ significantly in thermodynamic stability and formulation requirements (Souto et al., 2022). Nanoemulsions are kinetically stable emulsions with a droplet size of approximately 20–100 nm. They are usually created by high-energy methods like sonication and microfluidization. Likewise, microemulsions are 50–400 nm in size. They are thermodynamically stable and are formed spontaneously with surfactant or cosurfactant systems (Musakhanian and Osborne, 2025).

The internal oil phase of these systems allows solubilization of lipophilic cosmetic actives at high loadings. The small droplet size significantly increases the interaction of surface area with skin. By hydration of the stratum corneum and disarrangement of lipid packing, NEs/MEs enhance penetration of actives. Nanoscale emulsions are also used to stabilize sensitive ingredients by prevention of oxidation of oils and improving product textures (Deveci, 2025). Despite their benefits, challenges include potential irritation from high surfactant levels and to ensure long-term stability. Compared to polymeric or solid carriers, emulsions lack rigid matrix. So, they function more as transient carriers that merge with skin lipids. However, NEs and MEs are among most widely used nanotechnologies in over-the-counter skin products due to their simplicity and versatility (Souto et al., 2022).

### 3.4 Inorganic and hybrid nanomaterials

Inorganic nanomaterials bring unique optical, magnetic, and antimicrobial properties to aesthetics. Metallic nanoparticles such as gold and silver have attracted considerable attention in dermatological and cosmetic formulations due to their unique optical, antimicrobial, and anti-inflammatory properties (Raszewska-Famielec and Flieger, 2022). Gold nanoparticles (AuNPs) range from 5 to 50 nm in size and are incorporated into luxury anti-aging creams. AuNPs are inert and have antioxidant/anti-inflammatory effects. Some companies claim that they stimulate collagen and blood flow. A recent study noted that AuNPs (≈28 nm) in a cream remained stable and retained size/shape and suggested safe incorporation (Majerič et al., 2023). Silver NPs (10–100 nm) are valued for their broad-spectrum antimicrobial activity. They are used in acne products and wound-healing salves (Maeda et al., 2025). In addition to metallic nanoparticles, metal oxide nanomaterials play critical roles in aesthetic and dermatological formulations. Zinc oxide and titanium dioxide NPs with size range of approximately 20–100 nm are ubiquitous UV filters in sunscreens. They scatter and absorb UV light without causing the irritation of skin. Also, they mostly stay on the skin surface, minimizes penetration and are considered safe (Pan et al., 2021; Babarus et al., 2023).

Finally, natural nanomaterials such as nanoclay or nanofibrils (chitin nanofibers for wound healing) have cosmetic uses, and nanostructured fibrous materials can also form protective biointerfaces for biomedical applications (Wang et al., 2022). Hybrid carriers like polymer-lipid composites aim to combine these benefits (e.g., polymer-core/Lipid-shell NPs for targeted release). Each inorganic/hybrid system have own merits and demerits. In general, they offer various functions like photothermal ablation of fat, color-change, biosensing which are not possible with organic carriers. Contrary to this, they also raise more safety concerns like metal bioaccumulation and long-term toxicity (Morganti et al., 2023).

### 3.5 Comparative considerations in selecting nanocarrier platforms

Although numerous nanocarrier platforms have been developed for dermatological delivery, the selection of an appropriate system depends strongly on the physicochemical characteristics of the active compound, the intended depth of skin targeting, and the desired release profile. Lipid-based nanocarriers such as liposomes, solid lipid nanoparticles (SLNs), and nanostructured lipid carriers (NLCs) are widely used in cosmetic and dermatological formulations due to their high biocompatibility and structural similarity to the lipid matrix of the stratum corneum (Huang et al., 2024; Khan et al., 2023). These carriers can enhance dermal penetration by their interaction with epidermal lipids and by formation of an occlusive film that increases skin hydration and facilitates the diffusion of active compounds. Consequently, lipid-based systems are frequently used for delivery of antioxidants, vitamins, and anti-aging compounds in aesthetic dermatology (Geszke-Moritz and Moritz, 2016; Akombaetwa et al., 2023).

In contrast, polymeric nanoparticles provide greater structural stability and allow more precise control of drug release kinetics compared with lipid carriers (Beach et al., 2024; Madawi et al., 2023). Biodegradable polymers such as poly (lactic-co-glycolic acid) (PLGA) or chitosan can protect labile molecules from enzymatic degradation and provide sustained release profiles over extended periods (Mahar et al., 2023; Babić Radić et al., 2024). Additionally, polymeric nanocarriers can be surface-modified with targeting ligands or charged functional groups to improve interaction with skin cells or hair follicles, making them attractive for delivery of peptides, growth factors, and follicular therapies (Michniak-Kohn and Kohn, 2023; Andrade et al., 2025).

Emulsion-based nanocarriers, including nanoemulsions and microemulsions, are widely used in topical cosmetic products because of their high solubilization capacity and favorable sensory properties (Souto et al., 2022; Deveci, 2025). These systems enhance dermal penetration primarily by increasing drug solubilization and disrupting stratum corneum lipid packing; however, because they lack a rigid structural matrix, they typically function as transient delivery systems rather than long-term drug reservoirs (Musakhanian and Osborne, 2025).

Inorganic nanoparticles, such as titanium dioxide, zinc oxide, and metallic nanoparticles, provide distinct functional properties including UV protection, antimicrobial activity, and optical enhancement (Pan et al., 2021; Babarus et al., 2023; Majerič et al., 2023). Unlike organic nanocarriers, these materials are generally designed to remain on the skin surface or within the upper epidermal layers to minimize systemic exposure while providing protective or cosmetic functions (Araki and Baby, 2025). Therefore, the selection of a nanocarrier platform in aesthetic dermatology requires the balance of multiple factors including drug loading capacity, release kinetics, biocompatibility, penetration behavior, and safety profile.

## 4 Mechanisms of nanocarrier-enhanced dermal therapy

**FIGURE 3:**
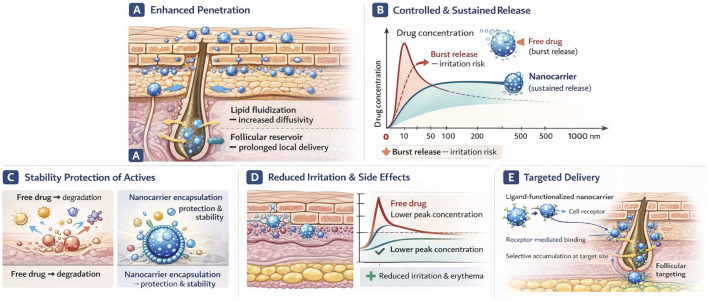
Figure 3 Schematic overview of the principal mechanisms underlying nanocarrier-based dermal therapy. These include **(A)** enhanced skin penetration and follicular deposition, **(B)** controlled and sustained drug release, **(C)** protection and stabilization of labile bioactive compounds through nanoencapsulation, **(D)** reduction of irritation and adverse effects through controlled drug exposure, and **(E)** targeted delivery via ligand-functionalized nanocarriers that selectively bind to receptors on skin cells or hair follicles to enable localized drug accumulation.

Building upon the penetration pathways and nanocarrier characteristics discussed in previous sections, nanocarriers improve aesthetic treatments through several key mechanisms summarized in Figure 3. They facilitate skin penetration and targeting. In addition they also provide controlled release, stabilize actives, and reduces side effects. These effects often overlap in certain conditions.

### 4.1 Enhanced skin penetration and targeting

Nanoparticles having size range of 10–200 nm can traverse skin via multiple routes more effectively than free molecules (Joseph et al., 2023). They exploit transappendageal (follicular) delivery. Different studies show that NPs preferentially deposit in hair follicles and release actives deep into the dermis. For example, 5% minoxidil formulated as 140 nm NPs delivered seven times more drug to the hair bulge than standard solution. This activated follicular stem cells and enhanced hair growth. The hair follicle acts as reservoir when they target hair or sebaceous glands (for acne or hair growth). Thus, colloidal carriers achieve far greater local concentration than creams (Oaku et al., 2022).

Additionally, nanocarriers can transiently convert the SC lipids into fluid. Ethanol, oleic acid, or nanoparticle components disturb packing of lipid, forms micro-pores and increase diffusivity. Lipid carriers (SLN/NLC) adhere to skin and form occlusive film that hydrates SC (swelling corneocytes) and forces drug passage. For instance, SLN have been shown to release corticosteroids that penetrate deeper than free drug, partly via SC lipid disruption (Geszke-Moritz and Moritz, 2016; Akombaetwa et al., 2023).

Surface chemistry also enables targeting. Cationic chitosan NPs bind more readily to slightly negatively charged skin surface and improves retention. Nanocarriers can be decorated with ligands (peptides, antibodies) that bind specific receptors on skin cells for localized action. Inorganic nanoparticles can convert external stimuli into therapeutic effects and have also been explored in theranostic applications combining targeted imaging and therapy for dermatological diseases (Raszewska-Famielec and Flieger, 2022; Zhang et al., 2022). The net result is that much higher amounts of active reach intended skin layer. Studies often report two to ten times higher dermal uptake versus conventional formulations (Oaku et al., 2022; Zazuli et al., 2025).

### 4.2 Controlled and sustained release

In addition to enhanced penetration, nanocarriers enable precise temporal control over drug release kinetics. Nanocarriers can encapsulate actives and release them slowly over time. They maintains therapeutic levels and reduces dosing frequency. Many polymeric NPs and lipid NPs are designed for sustained release. PLGA and PLA NP formulations degrade via hydrolysis and provide steady diffusion of drug. Similarly, LNCs and SLNs release lipophilic actives gradually as the lipid matrix erodes. In cosmetics, this translates to prolonged effect. A sunscreen loaded in SLNs can remain effective for hours longer, or an anti-wrinkle peptide in a liposome can exert its action over days (Venkatesan et al., 2025).

Embedded release also prevents sudden increase of drug that cause irritation. For instance, nanoencapsulated tretinoin or hydroquinone can deliver same dose as a cream but more gradually reduces redness (Zazuli et al., 2025). And by maintaining an active reservoir in skin, repeated re-application is less critical. Some smart formulations even allow stimulus-triggered release. The pH-sensitive nanogels release antioxidants when UV-induced acidity is high and give on-demand delivery (Kim, 2025).

### 4.3 Improved stability of bioactive compounds

Many cosmetic actives like vitamins C/E, retinoids, peptides and herbal extracts are unstable to light, oxygen, or pH. The encapsulation in nanoparticles can dramatically enhance their shelf-life and in-skin stability (Gupta et al., 2022). For example, vitamin C is easily oxidized in solution, but when packaged in a liposomal vesicle or polymer capsule, it remains active for long time (Favarin et al., 2022). Similarly, UV filters can be stabilized by dispersion in solid lipid matrix. Nanoparticles protect labile molecules from environmental degradation and enzymatic breakdown (Subroto et al., 2023).

Beyond ingredient stability, nanoparticles improve the physical properties of formulations. Nanoscale particles often yield transparent gels or lotions (as with nanoemulsions) and produce better aesthetics without sedimentation (Gupta et al., 2022). They can also enhance solubility as hydrophobic actives become miscible in lipid or polymer carriers. For example, a poorly soluble propolis extract was formulated into polymeric nanoparticles. It enabled its even distribution in anti-aging cream (An et al., 2022).

### 4.4 Reduction of adverse effects

Moreover, controlled delivery and encapsulation can mitigate irritation and systemic exposure associated with conventional formulations. For instance, targeting an antioxidant to melanocytes can reduce systemic exposure and allow lower total dose. One study suggested an improved safety profile, as liposomal tranexamic acid achieved comparable depigmentation efficacy to conventional hydroquinone treatment while reducing irritant erythema (Banihashemi et al., 2015; Zazuli et al., 2025). Encapsulation also allows replacement of harsh solvents and stabilizers. Instead of ethanol or propylene glycol, nanovesicles solubilize actives *in situ* (Majerič et al., 2023). Additionally, some carriers have intrinsic soothing effects:. The lipid nanoparticles hydrate skin (soothing eczema) and zinc oxide NPs calm irritation via their anti-inflammatory metal ions (Naveed et al., 2025).

However, not all side effects are eliminated. Some carriers like cationic dendrimers or surfactant-rich microemulsions can be irritating themselves. They can be mitigated by design strategies like PEGylation or by addition of antioxidants. In summary, nanotechnology often allows equal or greater efficacy at lower doses, with fewer excitations of non-targeted cells (Zazuli et al., 2025). In summary, nanotechnology-based formulations may enable comparable or improved therapeutic efficacy at lower doses while potentially reducing off-target effects; however, these outcomes can vary depending on nanocarrier composition, formulation design, and treatment context.

## 5 Clinical and skin medical aesthetic applications of nanotechnology

Nanocarriers have been applied to virtually all major cosmetic dermatology areas. Representative applications include anti-aging and skin rejuvenation, pigmentation disorders and whitening, acne and sebum regulation, hair restoration, and injectable nanomaterials. For each, nanotechnology offers unique benefits to deliver active therapies or perform new functions (Zhou et al., 2021).

### 5.1 Anti-aging and skin rejuvenation

Skin aging is characterized by wrinkles, loss of elasticity, and pigmentation changes. Nanocarriers amplify anti-aging treatments by co-delivering antioxidants, vitamins, and bioactive peptides. They not only improve appearance but also promote the health of skin. Key strategies include antioxidant delivery, stimulation of collagen, and removal of senescent cells. Nanotechnology has enabled delivery of classic anti-aging actives in more potent formats (Venkatesan et al., 2025).

#### 5.1.1 Antioxidants and vitamins

Oxidative stress from UV and pollution accelerates aging. Nanosystems can deliver high payloads of antioxidants. For example, coenzyme Q10 loaded into SLNs significantly penetrates the epidermis. Likewise, Q10-SLN improves skin hydration and reduces wrinkles more effectively than free Q10 emulsion (Atapour-Mashhad et al., 2024). Vitamin C nanoliposomes have been formulated for deep delivery. Vitamin E (tocopherol) in NLC/NE hydrogels also provides sustained antioxidant effect and moisture to dermis *in vivo* (Dias and Estevinho, 2025).

#### 5.1.2 Peptides and growth factors

Short peptides (matrixyl, argireline) and growth factors (EGF, FGF) stimulate collagen/elastin. Their use is hampered by poor skin penetration and degradation. Nanocarriers can transport intact peptides (Waszkielewicz and Mirosław, 2024). A biomimetic peptide-loaded cationic liposome can reduce the elastase activity by ∼50% *in vitro* and indicates inhibition of collagen breakdown (Venkatesan et al., 2025). Some companies use plasmid/lipid NPs to deliver genes encoding growth factors. Nanovesicles also enhance topical retinoids, as nanoencapsulated retinol shows less irritation with equal wrinkle reduction (Zhong et al., 2024).

### 5.2 Pigmentation disorders and skin whitening

In addition to anti-aging applications, nanotechnology-based systems have shown significant potential in managing pigmentary disorders. Hyperpigmentation (melasma, lentigines, post-inflammatory spots) is considered a major aesthetic concern. Treatments involve inhibition of melanin synthesis or dispersion of pigment. Nanocarriers permit more potent and targeted delivery of whitening agents. By increasing skin penetration and retention of melanin-inhibiting actives, they achieve faster and more uniform depigmentation (Singh et al., 2022).

#### 5.2.1 Melanin inhibitors in nanosystems

Antioxidants like kojic acid or arbutin have limited penetration. However, their skin levels can be enhanced by their encapsulation (Khan et al., 2023). For instance, a hesperidin (flavonoid) loaded NLC demonstrates strong anti-tyrosinase activity and inhibits melanin production better than free hesperidin. Menasome, a niosome-based formulation containing depigmenting agents such as kojic acid and arbutin, has been reported to enhance dermal permeation and improve the delivery efficiency of these melanin-inhibiting compounds. In cell culture, gallic acid in cationic niosomes suppresses tyrosinase and reduces melanin ∼45%. This evidence suggests that nanocarriers can enhance efficacy and reduce side-effects of traditional depigmenting agents (Venkatesan et al., 2025).

#### 5.2.2 Vitamin and brightening formulations

Niacinamide and vitamin C (ascorbic acid) are also used for brightening. Nano-liposomes deliver vitamin C deeply into the skin. Early clinical use has shown reduced blotchiness. A liposomal vitamin C/resveratrol/4-n-butylresorcinol cream produced visible depigmentation after 4 weeks. Photoreactive systems (nanoparticles that release O_2_ or ROS in melanosomes) are under research to selectively lighten pigment (Kwon et al., 2020).

### 5.3 Acne and sebum regulation

Similarly, nanoscale delivery systems have been explored for inflammatory and microbial skin conditions such as acne vulgaris. Acne vulgaris involves *Cutibacterium acnes* infection, inflammation, and overproduction of sebum. Traditional treatments like BPO, antibiotics, and retinoids have certain limitations. Nanotechnology offers new formulations to tackle bacterial biofilms, inflammation, and sebum control (Ivanova et al., 2023).

#### 5.3.1 Antimicrobials in nanocarriers

Silver nanoparticles (AgNPs) have broad-spectrum antibacterial effects and are used in some acne products (Reis et al., 2025). More innovatively, bio-based nanocapsules deliver botanical anti-acne agents. For example, zein (maize protein) nanocapsules loaded with oregano essential oil show smart release in acidic sebaceous conditions and completely inhibit *C. acnes* growth *in vitro*. These capsules also scavenged acne-associated ROS and protect skin cells. When they are incorporated into cream, the nano-EO penetrates into the epidermis and indicates real potential for acne therapy. Such systems combine antibacterial and anti-inflammatory action with controlled release (Ivanova et al., 2023).

#### 5.3.2 Retinoid and azelaic acid delivery

Isotretinoin (oral) causes systemic side effects and can cause irritation. Nano-carriers can deliver retinoids in milder and sustained forms. For instance, gold nanoshell-coated liposomes of retinol can reduce inflammatory acne lesions (photothermal effect kills bacteria and regulates glands). Azelaic acid (anti-inflammatory) in liposomes also shows improved follicular delivery (Tangau et al., 2022; Zhong et al., 2024).

#### 5.3.3 Sebum control

Nanocarriers are also used to deliver hormonal modulators or lipase inhibitors topically. For example, nanoparticle-stabilized formulations of benzoyl peroxide maintain high local concentration and can kill *C. acnes* with minimal diffusion into systemic circulation. Innovatively, the gold nanoparticles can be injected into fat to facilitate selective photothermolysis of sebaceous glands (called “NanoLipo” for fat/adipose reduction). Although this is primarily aimed at body contouring rather than facial acne (Sheng et al., 2014).

Overall, nanocarriers allow a combination of antimicrobial, anti-inflammatory, and hydration treatments in one regimen. It can potentially reduce long-term usage of antibiotic.

### 5.4 Hair restoration and scalp treatments

Beyond facial dermatological indications, nanotechnology has also demonstrated favorable applications in scalp and hair follicle therapies. Hair loss (androgenetic alopecia, telogen effluvium) is a major aesthetic complaint. Minoxidil and finasteride are standard topicals, but many patients find them only partially effective. Nanotechnology can enhance delivery of hair-growth stimulants and even introduce new modalities (Chen et al., 2020; Nagchowdhury and Patra, 2023).

#### 5.4.1 Nanoparticulate minoxidil

The landmark study showed that 5% minoxidil formulated as 140 nm nanoparticles (via bead milling) delivered the drug into the hair follicle stem cell 7.4-fold more than the commercial solution. The result was significantly greater hair regrowth in C57BL/6 mice (a model of androgenetic alopecia). The NPs penetrated deep into follicles and activated epithelial hair cells. This demonstrates that nano-MXD can potentially shorten treatment time and improve outcomes over standard formulations (Nagai et al., 2019).

#### 5.4.2 Lipidic and polymeric carriers

PLGA nanocapsules have been used to co-deliver finasteride and minoxidil to the scalp. For instance, PLGA/PEG mixed micelles (size ∼40–130 nm) encapsulating minoxidil showed enhanced follicular targeting and release. Solid lipid or nanostructured lipid carriers containing antioxidants (EGCG from green tea, coenzyme Q10) have been tested to reduce oxidative stress in the follicle microenvironment. Platelet-derived growth factors and stem cell exosome treatments are being formulated as nanovesicles for intradermal injection (Gupta et al., 2025).

#### 5.4.3 Novel approaches

Beyond actives, nanotechnology supports physical scalp therapies. For example, microparticles of gold or lipid are used as photothermal agents to locally ablate miniaturized hair follicles and stimulate new growth. It is somewhat analogous to laser hair regeneration. Also, nanofat (ultrafiltered adipose tissue or technically micro-sized fat cells) is injected into the scalp in experimental regenerative treatments. It provides stromal vascular fraction cells to promote hair follicle health (La Padula et al., 2023).

### 5.5 Nanotechnology in injectable aesthetic therapies

Injectable fillers and biostimulators are standard in aesthetic medicine. Nanotechnology is now entering this space as well, both by making improvements in existing injectables and introduction of novel therapies. While the majority of injectable aesthetic nanotherapy is still in early research phases, some clinical translations exist (nanofat, nano-hyaluronic acid, nanoparticle-assisted liposuction). These represent a frontier by combination of nanotechnology with plastic surgery and dermatology to achieve minimally invasive rejuvenation (Dey et al., 2026).

#### 5.5.1 Nanoparticle-enhanced lipolysis

A recent advancement in this field is NanoLipo. These are gold nanoparticles mixed into tumescent fluid and injected into adipose tissue. These are then illuminated with a specific laser wavelength. Gold nanoparticles convert absorbed laser energy into localized heat that selectively disrupts adipocytes while minimizing thermal damage to surrounding tissues such as blood vessels, connective tissue, and skin structures. This targeted photothermal effect differs from conventional liposuction, which mechanically removes fat and may produce more diffuse tissue trauma. A study showed that NanoLipo removed twice as much fat in 4 min (vs. standard liposuction time of 10 min) with fewer sessions. It is primarily a surgical aid but this approach exemplifies the injectable nanomedicine for body contouring (Sheng et al., 2014).

#### 5.5.2 Nanofat grafting

Nanofat is used intradermally for skin rejuvenation. Though these fat particles (∼100 µm) are above true nanoscale, they serve as a cell-rich nanotechnology as they deliver mesenchymal stromal cells and growth factors into the dermis. Different studies report improvement in wrinkles, scarring, and alopecia. Research is underway to refine this into standardized injectable microparticle systems (La Padula et al., 2023).

#### 5.5.3 Nanoscale fillers and regenerative scaffolds

Experimental nano-fillers like nano-hydroxyapatite or silica particles are being developed for volume filling. In tissue engineering, collagen or hyaluronan is crosslinked into nanoscale fibrils for injection, while protein-based functional hydrogels are also being explored for skin repair and regenerative applications (Lu et al., 2025). Likewise, injectable nanoporous hydrogels (e.g., alginate or PEG nanogels) loaded with fibroblast growth factor are used for scar remodeling, and recent studies highlight how mechanically responsive hydrogel networks can further improve structural stability and tissue integration (Liu et al., 2025). Magnetic NPs have also been explored to reshape tissue under external magnets for contouring. However it is still in preclinical phase (Elsapagh et al., 2025).

## 6 Clinical evidence and translational progress

Although extensive preclinical research has demonstrated the potential of nanotechnology-based delivery systems for dermatological applications, translation into clinical practice requires robust validation through well-designed experimental and clinical studies. Table 1 summarizes representative *in vitro*, *in vivo*, and early clinical investigations evaluating nanocarrier-based interventions in aesthetic dermatology.

**TABLE 1:** Key preclinical and clinical evidence supporting the translational application of nanotechnology-based systems in aesthetic dermatology.

Evidence level	Nanotechnology-based intervention	Model/Study design	Primary translational outcome	Comparative performance vs. conventional therapy	Reference
Preclinical	Lipid nanosystems (SLN, NLC)	*In vitro* human skin models (Franz diffusion cells)	2–4-fold increase in epidermal drug uptake with confirmed follicular and intercellular deposition	Superior skin penetration compared with standard creams and solutions	Hendawy et al. (2023)
Preclinical	SLN loaded with botanical antioxidants	Murine UV-induced photoaging model	Preservation of dermal collagen and elastin architecture following UV exposure	Improved anti-aging protection relative to free antioxidant formulations	Jeon et al. (2013)
Preclinical	Curcumin-loaded NLC	Imiquimod-induced rat psoriasis model	Reduced inflammatory markers and normalization of epidermal histology	Greater therapeutic efficacy than free curcumin	Shrotriya et al. (2018), Rani et al. (2025)
Preclinical	Metal oxide nanoparticles (TiO_2_, ZnO)	Mini-pig skin model	Effective UV protection without detectable systemic metal absorption	Comparable SPF with improved cosmetic elegance and safety	Maeda et al. (2025)
Preclinical	Minoxidil nanoparticles	C57BL/6 mouse alopecia model	Accelerated hair regrowth and increased follicular density	Superior hair growth response compared with conventional minoxidil solution	Oaku et al. (2022)
Clinical	Nano-hyaluronic acid; polymeric propolis nanoparticles	Randomized controlled trials (n ≈ 50)	Significant wrinkle reduction (∼20%) and improved skin hydration	Improved outcomes compared with placebo and base creams	An et al. (2022)
Clinical	Liposomal tranexamic acid; liposomal 4-butylresorcinol + resveratrol	Double-blind melasma trials (n ≈ 20)	∼30% reduction in MASI score with visible skin lightening in most patients	Comparable efficacy to hydroquinone with reduced irritation	Banihashemi et al. (2015)
Clinical	Nano-emulsion benzoyl peroxide + tretinoin	Double-blind clinical trials (n = 45–60)	Enhanced lesion clearance with reduced skin dryness	Similar efficacy with improved tolerability	Samadi et al. (2022)
Clinical	Nano-minoxidil gel; melatonin-loaded NLC	Pilot human trials (n = 30–40)	∼25% increase in hair count and higher patient satisfaction	Improved efficacy and tolerability versus standard minoxidil	Oaku et al. (2022)
Clinical	Nanofat; gold nanoparticle-assisted liposuction (NanoLipo)	Case series and small clinical trials (n ≈ 12)	Approximately 20% greater fat reduction per session with transient edema	Enhanced fat removal efficiency compared with conventional techniques	Sheng et al. (2014)

A large proportion of the available evidence currently originates from *in vitro* cellular models and animal studies that investigate nanoparticle penetration, drug stability, antimicrobial activity, or anti-inflammatory effects. These studies are valuable for mechanistic understanding and formulation optimization but do not always directly translate to clinical efficacy in humans. Animal models, such as murine models used to evaluate follicular delivery of nanoparticle-formulated minoxidil or antioxidant-loaded lipid nanoparticles, provide important insights into pharmacokinetics and follicular targeting; however, differences in skin structure between animal models and human skin must be considered when interpreting these results.

Several early clinical investigations have provided encouraging results. For example, clinical studies evaluating nano-hyaluronic acid formulations or liposomal delivery systems have reported improvements in skin hydration, wrinkle reduction, and overall skin appearance with favorable tolerability profiles (Jegasothy et al., 2014; An et al., 2022). These human investigations, although limited in number, provide important early clinical evidence demonstrating improvements in measurable dermatological outcomes such as skin hydration, wrinkle depth, pigmentation indices, and patient-reported aesthetic satisfaction. In pigmentary disorders, studies which examine liposomal or nanoparticle-based depigmenting agents have demonstrated measurable reductions in melasma severity indices when they are compared with conventional topical formulations (Banihashemi et al., 2015; Kwon et al., 2020). Recent research has also explored the integration of nanotechnology with drug repurposing strategies to improve the topical treatment of skin diseases, including nanoparticle-based delivery systems designed to enhance the therapeutic efficacy and targeting of anticancer agents while reducing systemic toxicity (Martins et al., 2025). However, these emerging studies also highlight several limitations, including variability in nanocarrier composition, differences in particle size distribution, and heterogeneity in formulation design, which complicate direct comparison of clinical outcomes across studies and emphasize the need for standardized evaluation protocols.

Another important consideration is the robustness of study design. Many reported investigations involve small sample sizes, short treatment durations, or open-label study designs. While these early-phase studies provide valuable proof-of-concept evidence, larger randomized controlled trials with standardized outcome measures will be necessary to confirm long-term efficacy and safety. In addition, variability in nanoparticle composition, size distribution, and formulation methods complicates direct comparison between studies.

Despite these limitations, the cumulative evidence suggests that nanocarrier-based delivery systems can enhance dermal drug deposition, improve stability of active compounds, and potentially reduce adverse effects compared with conventional topical formulations. Continued integration of mechanistic research with well-designed clinical studies will be essential to facilitate the successful translation of nanotechnology-based therapies into routine aesthetic dermatological practice.

## 7 Safety, toxicology, and biocompatibility considerations

While nanotechnology offers many benefits, the safety is also of paramount importance. Nanoparticles can interact with biological systems in unexpected ways because of their small size and large surface area. Key concerns in aesthetic applications include local skin toxicity, immune/inflammatory responses, systemic absorption, and long-term effects (Zazuli et al., 2025). Key safety concerns and risk-mitigation strategies associated with nanocarrier systems used in aesthetic dermatology are summarized in Table 2.

**TABLE 2:** Safety considerations and risk-mitigation strategies for nanocarrier systems used in aesthetic dermatology.

Nanocarrier category	Potential safety concern	Key risk factors	Risk-mitigation strategies	Clinical relevance	References
Lipid-based nanocarriers (liposomes, SLN, NLC)	Skin irritation at high lipid or surfactant concentrations	Particle size, lipid crystallinity, surfactant type	Use of biocompatible lipids; optimized surfactant levels; controlled particle size (50–300 nm)	Generally well tolerated; widely used in cosmetic and clinical products	Erduran et al. (2024)
Polymeric nanoparticles (PLGA, chitosan, HA)	Inflammatory response or delayed clearance	Polymer composition, degradation rate, surface charge	Selection of biodegradable polymers; PEGylation; surface neutralization	Suitable for sustained delivery with low systemic exposure	Nunes et al. (2022)
Dendrimers	Cytotoxicity and membrane disruption	Small size (<20 nm), high cationic surface charge	Surface modification (PEGylation, acetylation); dose limitation	Promising but requires careful design and safety validation	Janaszewska et al. (2019)
Nanoemulsions/Microemulsions	Skin irritation and barrier disruption	High surfactant or co-surfactant content	Use of mild surfactants; optimization of oil/surfactant ratios	Common in cosmetics; tolerability depends on formulation	Souto et al. (2022)
Metal oxide nanoparticles (ZnO, TiO_2_)	Oxidative stress, photocatalytic activity	Particle coating, UV exposure, aggregation state	Surface coating (silica, alumina); controlled particle size; photostability testing	Considered safe for sunscreens with minimal systemic absorption	Pan et al. (2021)
Metallic nanoparticles (Au, Ag)	Long-term accumulation, oxidative stress	Size, dose, exposure duration	Low-dose topical use; surface functionalization; restricted clinical indications	Mainly used for antimicrobial or procedural applications	Pan et al. (2021)
Follicular-targeted nanocarriers	Potential systemic uptake via follicles	Very small particle size, compromised skin barrier	Size control (>20 nm); localized delivery; avoidance on damaged skin	Enhances targeting while minimizing systemic exposure	Andrade et al. (2025)
Injectable nanotechnologies (NanoLipo, nanofat)	Transient inflammation or edema	Injection technique, nanoparticle concentration	Controlled dosing; procedural standardization; clinical monitoring	Applied in medical aesthetic procedures under professional supervision	Sheng et al. (2014)

### 7.1 Skin and systemic toxicity

Most lipid-based carriers (phospholipids, fatty acids) and biopolymers (HA, chitosan) are well-tolerated on skin. They biodegrade into non-toxic metabolites like fatty acids and lactic acid. Even metal oxide NPs (ZnO, TiO_2_) largely remain on the stratum corneum and are generally recognized as safe at cosmetic doses (Erduran et al., 2024). However, there are certain factors which affect the toxicity. These include particle size, concentration, shape, surface charge, and formulation components (Awashra and Młynarz, 2023).

Firstly, particle size plays importance role in topxicity. The nanoparticle with size less than 10 nm might penetrate beyond dermis into circulation. In practice, most cosmetic NPs are larger than 20 nm and tend to remain confined to skin layers. But still, systemic uptake of some fraction is possible, especially via follicles or compromised skin (Maeda et al., 2025). Secondly, composition and surface chemistry also varies. Cationic dendrimers or cationic surfactants can cause cell membrane disruption and inflammation. Surfactant-heavy formulations (like some microemulsions) can defat and irritate skin. In contrast, PEGylated or lipid-coated particles are inert. Thus, it is better to use inherently safe materials and avoid bioactive surfactants where possible (Venkatesan et al., 2025). Thirdly, high concentrations of NPs may overload clearance mechanisms. Chronic exposure to TiO_2_ or silver NPs can raise risks of ROS generation or argyria-like effects. At the cellular level, nanoparticle-associated toxicity is frequently linked to oxidative stress mechanisms, including excessive generation of reactive oxygen species (ROS), mitochondrial dysfunction, lipid peroxidation, and activation of pro-inflammatory signaling pathways such as NF-κB and MAPK, which may contribute to local inflammatory responses or cellular damage under prolonged exposure conditions (Xu et al., 2023).

If NPs enter circulation (through follicular capillaries or broken skin), they distribute to organs (liver, kidney, spleen) based on physicochemical properties and can cause systemic toxicity. Gold NPs may accumulate in liver and silver NPs can induce oxidative stress in cells. However, dermal absorption rates are usually low. No major systemic toxicities have been reported for commercially used nanocosmetics in humans. Safety assessments (cytotoxicity assays, skin sensitization tests, animal studies) are crucial for development of new products (Bhardwaj et al., 2023; Maeda et al., 2025).

### 7.2 Immunological and inflammatory responses

The skin’s immune system may recognize nanoparticles as foreign. Langerhans cells in the epidermis can take up NPs and potentially present their components to T cells. Some NPs (especially cationic ones) can activate inflammasomes or complement. This could manifest as contact dermatitis and worsen inflammatory conditions if not checked. However, many nanomaterials are designed to minimize such responses. PEGylated liposomes evade phagocytosis to an extent. Studies of topical NLCs and SLNs have generally shown minimal erythema or irritation in human trials. The nano-hyaluronic acid study reported no adverse events in patients. The nano-propolis and nano-HA trials also noted very good tolerance (Jegasothy et al., 2014; Madawi et al., 2023).

However, prolonged or high-dose exposure might unbalance redox or immune homeostasis. It is recommended that nano-aesthetic products should avoid triggers of inflammatory pathways. They should be involved in reducing reactive surfaces (e.g., oxide groups), avoiding high positive charges, and using antioxidants in formulations (Zazuli et al., 2025).

## 8 Regulatory landscape and standardization challenges

Nanotechnology implementation in cosmetics and aesthetic medicine touches upon various regulatory areas that form a multifaceted environment. Nanoscale ingredients are now regulated in most jurisdictions within the existing cosmetic, pharmaceutical, or medical device regulations. They are usually devoid of nano-specific legal definitions or binding arrangements. As a result, regulatory guidance has developed incrementally rather than harmonized global standards (Kumud and Sanju, 2018; Chandran et al., 2024).

### 8.1 United States (FDA)

Cosmetics of nanoparticles are not pre-approved. In 2014, the U.S. Food and Drug Administration (FDA) published Guidance for Industry: Safety of Nanomaterials in Cosmetic Products. It is a non-binding guideline, which suggests that nanomaterials should be characterized (size, shape, surface) and toxicologically tested (dermal, systemic). It focuses on the consideration of the altered properties of NPs (e.g., reactivity). Even though adherence to this directive is optional, the FDA still has the mandate to exercise regulatory measures in case a product is discovered to be unsafe or misbranded. It is important to note that FDA lacks a legally defined meaning of nanomaterial, with a working size range of around less than 100 nm mentioned, but functionality and properties are considered more significant than size (Kumud and Sanju, 2018; Katz et al., 2022).

### 8.2 European Union

The SCCS (Scientific Committee on Consumer Safety) must approve all the nanomaterials used in cosmetics and list them in the Cosmetic Regulation Annex. These products should indicate ingredients as nano on the package (e.g., titanium dioxide (nano) on package). Moreover, European Commission has publicly available list of the nanomaterials used in cosmetics that increases consumer and professional transparency. The SCCS has issued comprehensive information on safety evaluation of nanomaterials that includes routes of exposure, toxicokinetics, and exposure risk. In addition to legislation particular to cosmetics, the nanomaterials are also subject to the Registration, Evaluation, Authorization and Restriction of Chemicals (REACH) regulation. They demand that manufacturers and importers to submit safety data of nanoforms of substances. In general, EU structure provides greater control and transparency of consumers compared to other areas (Henkler et al., 2012; Gautam et al., 2022).

### 8.3 Other regions

Japan, Canada and Australia adopt the same precautionary measures. At the global level, the International Cooperation on Cosmetics Regulation (ICCR), a collaborative forum involving regulatory authorities from the European Union, the United States, Canada, Japan, and Brazil, has issued consensus principles regarding the definition, labeling considerations, and safety evaluation strategies for nanotechnology in cosmetic products (Xiankai et al., 2021).

Overall, regulatory approaches toward nanomaterials differ across major jurisdictions. In the United States, regulatory oversight primarily relies on post-market monitoring and manufacturer responsibility, supported by non-binding FDA guidance on nanomaterial safety assessment. In contrast, the European Union applies a more precautionary regulatory framework that requires pre-market safety evaluation of nanomaterials by the Scientific Committee on Consumer Safety (SCCS) and mandatory labeling of nano-ingredients in cosmetic products. Other regions, including Japan and Canada, generally adopt precautionary approaches that align more closely with EU regulatory principles while still relying on existing cosmetic or pharmaceutical regulatory structures. These differences highlight the need for greater international harmonization of safety evaluation standards and regulatory definitions for nanotechnology-based dermatological products (Chandran et al., 2024; Gautam et al., 2022).

Even with such efforts, there are still great regulatory and standardization challenges that exist. Nanomaterials are not globally harmonized in definition. Certain of these frameworks are based on firm size alone, others take into account properties or biological behavior. Organizations like OECD and ISO still develop standardized methods of testing the safety of nanomaterials, and most of the methods are not fully validated. Besides, aesthetic nanomedicines (e.g., drugs or devices with nanoparticles) are not regulated as cosmetics, but as drugs or devices. These products must undergo careful preclinical development, clinical testing, and regulatory representation like conventional medicines or products. Such applications have been specifically considered by regulatory organs such as the FDA nanotechnology task forces because they have higher risk profiles (Thakur and Bala, 2025).

Manufacturers developing aesthetic nanoproducts are expected to exercise substantial due diligence, employing advanced analytical techniques such as dynamic light scattering (DLS), transmission electron microscopy (TEM), stability profiling, and comprehensive toxicological testing (including genotoxicity and sensitization). In practice, however, regulatory enforcement often lags behind technological innovation. As a result, consumers and clinicians should look for products supported by quality safety data (Monika et al., 2025).

## 9 Future directions and emerging trends

Nanotechnology in aesthetic dermatology is rapidly advancing toward smarter, more personalized, and clinically integrated applications. These applications include stimuli-responsive nanocarriers, targeted bio-nano systems, theranostic platforms, advanced delivery devices such as nanoparticle-loaded microneedles, and scalable manufacturing approaches driven by microfluidics, 3D printing, and AI-guided formulation design (Pareek et al., 2026). These emerging trends promise highly controlled, customized, and multifunctional nanocosmetic therapies. However, significant challenges still remains. These include limited long-term safety data, uncertainties in nanoparticle penetration and dosimetry, gaps in clinical translation and large-scale trials, regulatory inconsistencies, high production costs, and the need for interdisciplinary collaboration. These knowledge gaps need to be addressed through standardized testing, regulatory harmonization, cost-effective manufacturing. Likewise rigorous clinical validation will be essential to ensure the safe, effective, and ethical integration of nanotechnology into cosmetic and aesthetic medicine.

## 10 Conclusion

Nanotechnology is reshaping medical aesthetic dermatology by improving the delivery, stability, and effectiveness of cosmetic and therapeutic agents. Nanoscale carriers enhance skin penetration, enable controlled release, and reduce adverse effects, leading to improved outcomes in anti-aging, pigmentation, acne, hair restoration, and injectable aesthetic treatments. Although early clinical studies show promising efficacy and safety, challenges remain regarding long-term toxicity, regulatory standardization, and large-scale clinical validation. Continued research, rigorous clinical trials, and harmonized regulatory frameworks are essential to ensure safe and effective integration of nanotechnology into aesthetic medicine.
